# Pancreatic metastases from ocular malignant melanoma: the use of endoscopic ultrasound-guided fine-needle aspiration to establish a definitive cytologic diagnosis: a case report

**DOI:** 10.1186/s13256-016-1121-2

**Published:** 2016-12-01

**Authors:** Diogo Turiani Hourneaux De Moura, Danielle Azevedo Chacon, Ryan Tanigawa, Martin Coronel, Spencer Cheng, Éverson L. A. Artifon, José Jukemura, Eduardo Guimarães Hourneaux De Moura

**Affiliations:** 1 Gastrointestinal Endoscopy Unit, Hospital das Clínicas da Faculdade de Medicina da Universidade de São Paulo, Av. Dr Enéas de Carvalho Aguiar, 225, 6° andar, bloco 3, Cerqueira Cezar, ZIP Code 05403-010 São Paulo, SP Brazil; 2 Patology Unit, Hospital das Clínicas da Faculdade de Medicina da Universidade de São Paulo, Av. Dr Enéas de Carvalho Aguiar, 225, Andar, bloco, Cerqueira Cezar, ZIP Code 05403-010 São Paulo, SP Brazil; 3 Gastrointestinal Surgery Unit, Hospital das Clínicas da Faculdade de Medicina da Universidade de São Paulo, Av. Dr Enéas de Carvalho Aguiar, 225, andar, bloco, Cerqueira Cezar, ZIP Code 05403-010 São Paulo, SP Brazil

**Keywords:** Endoscopic ultrasound, Endoscopic ultrasound-guided fine-needle aspiration, Melanoma, Uveal melanoma, Metastatic melanoma, Pancreatic metastasis, Case report

## Abstract

**Background:**

When encountering solid pancreatic lesions, nonpancreatic primary metastases are rare and differentiating a metastasis from a primary neoplastic lesion is challenging. The clinical presentation and radiologic features can be similar and the possibility of a pancreatic metastasis should be considered when the patient refers to a history of a different primary cancer. Endoscopic ultrasound offers a key anatomical advantage in accessing the pancreas and endoscopic ultrasound-guided fine-needle aspiration has become the gold standard method for diagnosing pancreatic lesions.

**Case presentation:**

A 58-year-old white Hispanic woman with a history of uveal malignant melanoma, presented with abdominal pain and jaundice. On admission, laboratory tests were performed (her total bilirubin was 6.37 mg/dL with a direct fraction of 5.30 mg/dL). Cross-sectional, abdominal computed tomography with contrast, showed a low-attenuating lesion localized in the pancreatic head (measuring 4 × 3 cm) and a thinner section of the distal bile duct suspicious for compression. Our patient was scheduled for an endoscopic ultrasound-guided fine-needle aspiration to establish a diagnosis. Endoscopic ultrasound showed a solid, hypoechoic, well-defined lesion with regular contours (measuring 3.17 × 2.61 cm), localized between the head and neck of the pancreas. Endoscopic ultrasound-guided fine-needle aspiration was performed with a 22G needle and cytology confirmed the diagnosis of metastatic melanoma. Our patient subsequently underwent right orbital exenteration, followed by duodenopancreatectomy without complications. At the moment our patient is receiving adjuvant chemotherapy at an outside oncology clinic.

**Conclusions:**

To the best of our knowledge, this is a very rare presentation of an ocular malignant melanoma with an isolated pancreatic metastasis causing symptomatic biliary obstruction. Endoscopic ultrasound-guided fine-needle aspiration has proven to be the best method to diagnose solid pancreatic lesions. In this particular case, cytology was essential in confirming the diagnosis and guiding the most adequate therapy, which was a pancreatic resection, ocular exenteration of the melanoma, followed by adjuvant chemotherapy.

## Background

Malignant pancreatic lesions generally arise from the gland itself. Nonpancreatic primary metastases are rare but usually seen in patients with widely metastatic disease [1]. The frequency of this finding is variable, from 4.5 % in case series up to 11 % in data from autopsies. Isolated pancreatic metastasis can be found in only 2 % of cases [2–4].

The most common malignancies that metastasize to the pancreas are: renal, lung, breast, and colon cancer, followed by sarcoma and melanoma [5, 6]. Malignant melanoma commonly metastasizes to the gastrointestinal tract. Interestingly, autopsy-derived data demonstrates that 50 to 60 % of metastatic malignant melanoma patients can have gastrointestinal metastases but the clinical diagnosis is made only in 1.5 to 4.4 % of patients [7].

Metastatic malignant melanoma usually affects multiple sites. Isolated organ metastases are unusual and isolated pancreatic metastases are extremely rare. Uveal malignant melanoma is the most common primary intraocular malignancy. The majority of patients present with visual complaints, but as part of their primary workup, abdominal cross-sectional imaging should be performed since the primary metastatic location is the liver, in over 90 % of cases [8]. We found only one case report of a patient with metastatic uveal melanoma where the lesion spread to the gallbladder and head of the pancreas [9].

Endoscopic ultrasound (EUS) provides us with high-quality images to look at the pancreas and nearby structures and the linear array echo endoscope has a working channel that allows us to accurately target different areas of the pancreas with minimal trauma to surrounding tissues. For this reason, endoscopic ultrasound-guided fine-needle aspiration (EUS-FNA) is now the gold standard method for sampling the pancreas. In the particular case of pancreatic metastasis, EUS-FNA has shown to be very useful, since establishing a diagnosis with cross-sectional imaging alone is not possible and this method offers the advantage of real-time tissue acquisition for diagnostic confirmation. This is of particular importance for identifying adequate surgical candidates, for avoiding unnecessary surgeries, and helping triage nonsurgical patients for less invasive procedures [10, 11].

Since this is a rare finding, there is a scarce amount of data addressing the surgical management of these pancreatic metastases. Patients who have isolated nonpancreatic primary metastases have shown to have excellent surgical outcomes and long-term survival can be achieved in patients who have these lesions resected [4, 12, 13].

## Case presentation

**Fig. 1:**
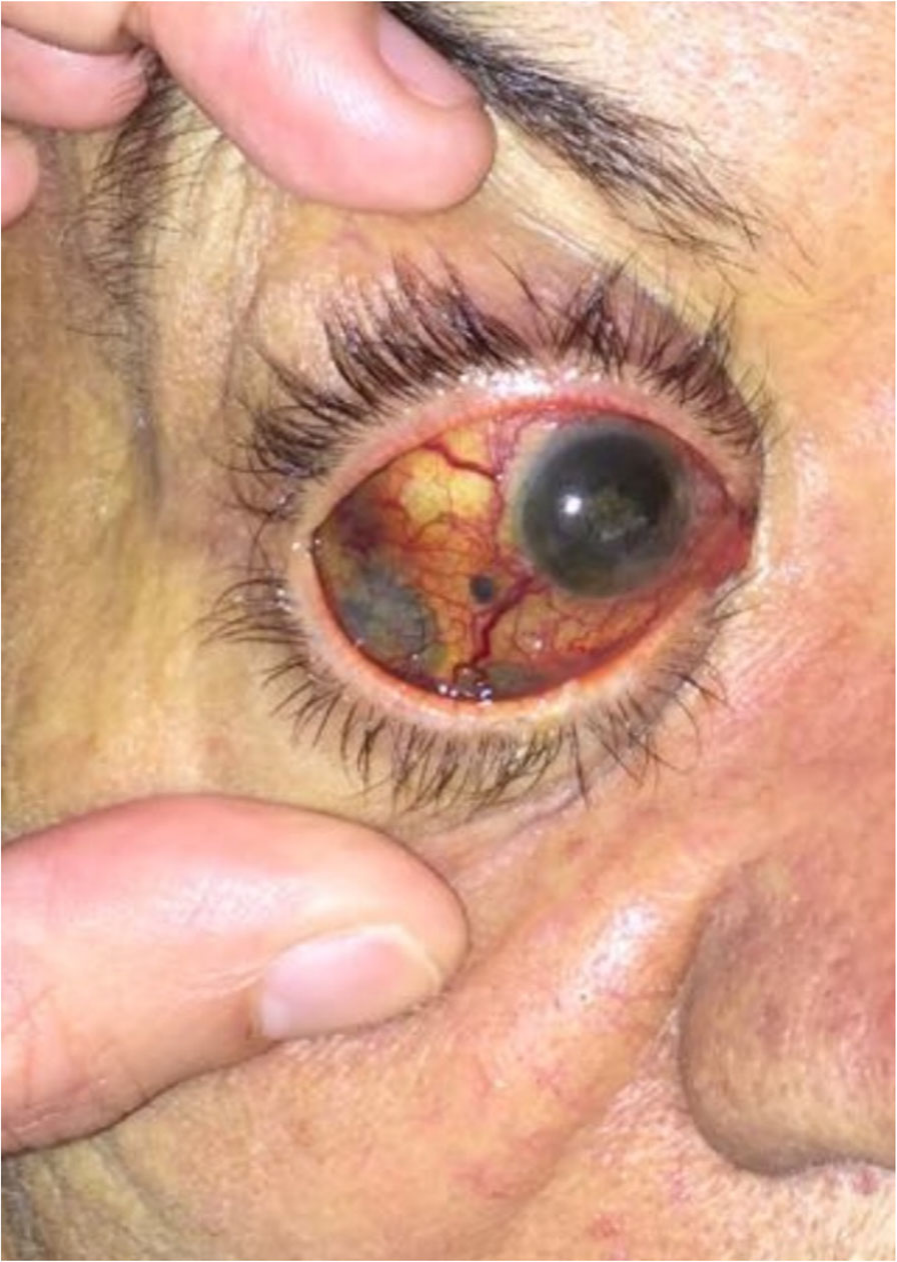
Right eye uveal melanoma showing dark brown pigments with diffuse extrascleral extension

This is the case of a 58-year-old white Hispanic woman with a history of uveal melanoma in her right eye (Fig. 1). She was admitted to the hospital with jaundice and abdominal pain for 10 days. On admission, laboratory tests were obtained (a complete blood count was within normal limits, amylase: 136 U/L, total bilirubin: 6.37 mg/dL with a direct fraction of 5.30 mg/dL). Cross-sectional, abdominal computed tomography (CT) with contrast, showed a low-attenuating lesion localized in the pancreatic head (measuring 4 × 3 cm) and a thinner section of the distal bile duct suspicious for compression).

**Fig. 2:**
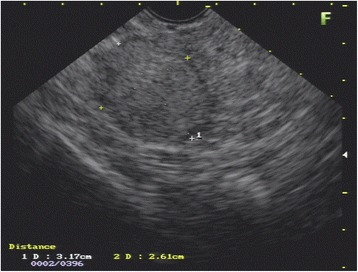
Endoscopic ultrasound scan showing a solid, heteroechoic with predominantly hypoechoic areas, well-defined lesion with regular contours (measuring 3.1 × 2.6 cm), localized between the head and neck of the pancreas

After a multidisciplinary meeting, our patient was scheduled for EUS-FNA. EUS showed a solid, heteroechoic with predominantly hypoechoic areas, well-defined lesion with regular contours (measuring 3.1 × 2.6 cm), localized between the head and neck of the pancreas (Fig. 2). There was no vascular or lymph node invasion identified. EUS-FNA was performed with a 22G needle using the fanning technique.

**Fig. 3:**
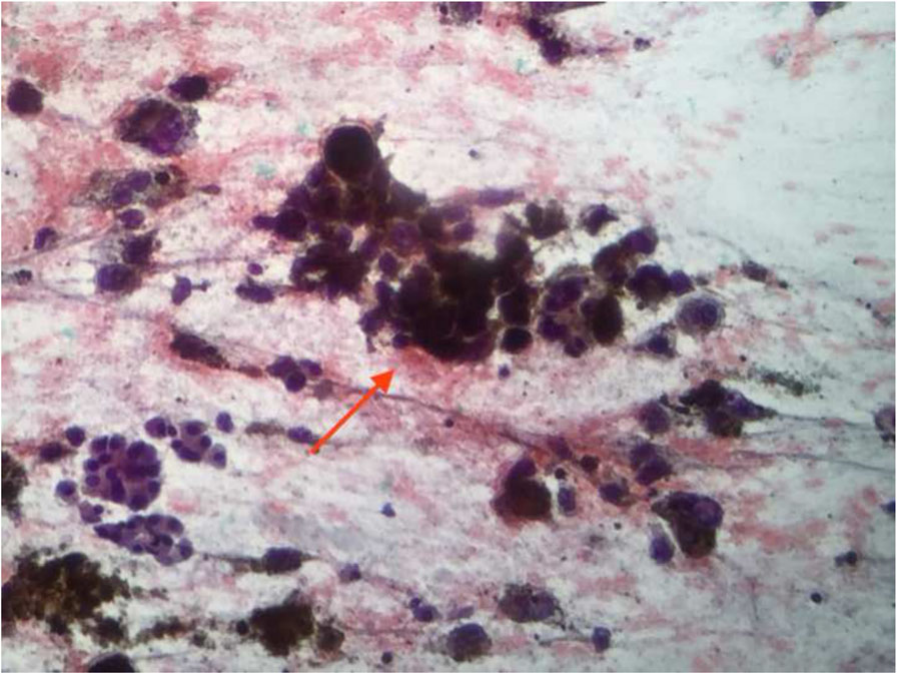
Endoscopic ultrasound-guided fine-needle aspiration specimen. Cytology shows markedly cells with a discohesive dispersed pattern, with variation in size and shape including epithelioid and spindle-shaped cells with plasmacytoid and round nuclei. *Arrow* showing the nuclear chromatin is clumping and irregular with excessive parachromatin clearing, there are single or multiple macronucleoli, and abundant cytoplasm with deep brownish-black granules obscuring the cell details. The background shows necrosis and hemorrhage

In the cytology specimens (Fig. 3), the cells presented have a discohesive dispersed pattern, with marked variation in size and shape including epithelioid and spindle-shaped cells with plasmacytoid and round nuclei. The nuclear chromatin is clumping and irregular with excessive parachromatin clearing, there are single or multiple macronucleoli, and abundant cytoplasm with deep brownish-black granules obscuring the cell details. The background shows necrosis and hemorrhage.

**Fig. 4:**
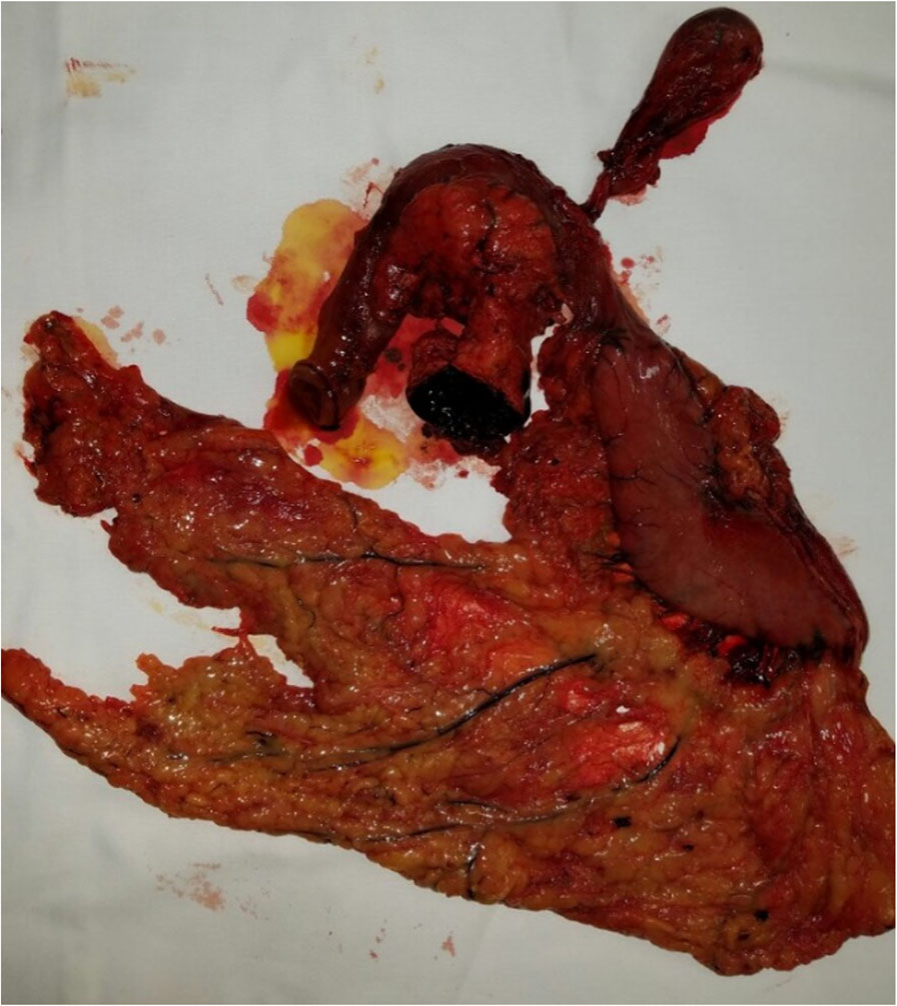
Surgical specimen after duodenopancreatectomy

Our patient underwent right orbital exenteration and followed by duodenopancreatectomy (the Whipple procedure), without any complications (Fig. 4). The surgical specimen showed undifferentiated cells, with extended disposition of brown pigment, infiltrating the pancreas and the serosa of the duodenal wall. Immunohistochemistry was positive for Melan-A, HMB45, vimentin, S-100 protein and negative for cytokeratin, all consistent with metastatic malignant melanoma. At the moment, our patient is receiving adjuvant chemotherapy at an outside oncology clinic.

## Discussion

Most of the solid pancreatic tumors are primary in origin and nonprimary pancreatic metastases are very rare [1], with just a few reported cases in the literature. The most common primary locations are kidney, lung, and breast [5, 6]. In this particular case, we identified a patient with a previous uveal melanoma that had an isolated pancreatic metastasis.

In general, over 50 % of patients with pancreatic metastases are discovered incidentally during imaging studies, only the minority of patients with pancreatic metastases present with symptoms of malignant biliary obstruction (jaundice, abdominal pain, and weight loss) making this a challenging clinical scenario [14, 15].

When evaluating solid pancreatic lesions, computed tomography (CT), magnetic resonance imaging (MRI), and EUS are very sensitive diagnostic methods, however, EUS allows for sampling, making this the preferred method to secure a diagnosis [14].

Studies have reported a sensitivity of 87–100 % using endoscopic ultrasound in detecting solid pancreatic lesions. Also, provides a high negative predictive value in ruling out pancreatic masses in the case these are not identified by EUS. [16]. When compared to CT, MRI and EUS have a better performance in detecting small lesions, especially if they are less than 3 mm in diameter both within the pancreas and in the duodenal wall [17].

As for the appearance of pancreatic metastasis in EUS, they are hypoechoic and usually are well-defined with regular margins, [6] with similar characteristics to neuroendocrine tumors [18].

To be able to distinguish a primary pancreatic tumor from a nonpancreatic metastasis, pancreatic sampling is critical. Aspirates of melanoma are cellular and consist of discohesive malignant-appearing cells with nuclear pleomorphism and prominent nucleoli. The admixture of malignant epithelioid and spindle-shaped cells and the presence of melanin pigment are helpful clues to establishing the diagnosis. Confirmatory immunostaining was demonstrating immunoreactivity for some or all of melanoma markers, such as S100, HMB-45, Melan-A and micropthalmia transcription factor (MITF), can be used to aid the diagnosis of metastatic melanoma [19].

This case report illustrates an interesting and rare clinical scenario of nonpancreatic primary metastases from uveal malignant melanoma causing malignant biliary obstruction. That was conclusively proven using EUS-FNA.

## Conclusions

Uveal malignant melanoma is the most common intraocular malignancy and it commonly metastasizes to the liver, in over 90 % of cases. This is a rare case report of an isolated nonpancreatic primary metastases. EUS-FNA is the gold standard method to characterize and diagnose solid pancreatic lesions, making this an invaluable tool to help guide the clinical management and decide a patient’s surgical candidacy. In carefully selected cases, the resection of isolated nonpancreatic primary metastases has shown to have good outcomes and improve the survival of these patients.

## Abbreviations

CT: computed tomography
EUS: endoscopic ultrasound
EUS-FNA: endoscopic ultrasound-guided fine-needle aspiration
MRI: magnetic resonance imaging

## References

[1] Eloubeidi MA, Tamhane AR, Buxbaum JL. Unusual, metastatic, or neuroendocrine tumor of the pancreas: a diagnosis with endoscopic ultrasound-guided fine-needle aspiration and immunohistochemistry. Saudi J Gastroenterol. 2012;18:99–105. 10.4103/1319-3767.93810 PMC3326984 22421714

[2] Waters L, Si Q, Caraway N, Mody D, Staerkel G, Sneige N. Secondary tumors of the pancreas diagnosed by eundoscopic ultrasound-guided fine-needle aspiration: a 10-year experience. Diagn Cytopathol. 2014;42(9):738–43. 10.1002/dc.23114 24554612

[3] Layfield LJ, Hirschowitz SL, Adler DG. Metastatic disease to the pancreas documented by endoscopic ultrasound guided fine-needle aspiration: a seven- year experience. Diagn Cytopathol. 2012;40(3):228–33. 10.1002/dc.21564 22334524

[4] Sperti C, Pasquali C, Liessi G, et al. Pancreatic resection for metastatic tumors to the pancreas. J Surg Oncol. 2003;83(3):161–6. 10.1002/jso.10262 12827684

[5] Goyal J, Lipson EJ, Rezaee N, et al. Surgical resection of malignant melanoma metastatic to the pancreas: case series and review of literature. J Gastrointest Cancer. 2012;43:431–6. 10.1007/s12029-011-9320-y PMC3742086 21912850

[6] Fusaroli P, d’Ercole MC, De Giorgio R, et al. Contrast harmonic endoscopic ultrasonography in the characterization of pancreatic metastases (with video). Pancreas. 2014;43:584–7. 10.1097/MPA.0000000000000081 24713844

[7] McLoughlin JM, Zager JS, Sondak VK, Berk LB. Treatment options for limited or symptomatic metastatic melanoma. Cancer Control. 2008;15(3):239–47. 10.1177/107327480801500307 18596676

[8] Chattopadhyay C, Kim DW, Gombos DS, Oba J, Qin Y, Williams MD, Esmaeli B, Grimm EA, Wargo JA, Woodman SE, Patel SP. Uveal melanoma: from diagnosis to treatment and the science in between. Cancer. 2016;122(15):2299–312. 10.1002/cncr.29727 PMC5567680 26991400

[9] Cunningham JD, Cirincione E, Ryan A, Canin-Endres J, Brower S. Indications for surgical resection of metastatic ocular melanoma. A case report and review of the literature. Int J Pancreatol. 1998;24(1):49–53. 10.1007/BF02787531 9746890

[10] Pang JC, Roh MH. Metastases to the pancreas encountered on endoscopic ultrasound-guided, fine-needle aspiration. Arch Pathol Lab Med. 2015;139(10):1248–52. 10.5858/arpa.2015-0200-RA 26414469

[11] DeWitt J, Jowell P, Leblanc J, et al. EUS-guided FNA of pancreatic metastases: a multicenter experience. Gastrointest Endosc. 2005;61:689–96. 10.1016/s0016-5107(05)00287-7 15855973

[12] Crippa S, Angelini C, Mussi C, et al. Surgical treatment of metastatic tumors to the pancreas: a single center experience and review of the literature. World J Surg. 2006;30(8):1536–42. 10.1007/s00268-005-0464-4 16847716

[13] Reddy S, Edil BH, Cameron JL, Pawlik TM, Herman JM, Gilson MM, Campbell KA, Schulick RD, Ahuja N, Wolfgang CL. Pancreatic resection of isolated metastases from nonpancreatic primary cancers. Ann Surg Oncol. 2008;15(11):3199–206. 10.1245/s10434-008-0140-7 18784960

[14] Triantopoulou C, Kolliakou E, Karoumpalis I, et al. Metastatic disease to the pancreas: an imaging challenge. Insights Imaging. 2012;3:165–72. 10.1007/s13244-011-0144-x PMC3314732 22696042

[15] Scatarige JC, Horton KM, Sheth S, et al. Pancreatic parenchymal metastases: observations on helical CT. AJR Am J Roentgenol. 2001;176:695–9. 10.2214/ajr.176.3.1760695 11222207

[16] Coronel E, Waxman I. State-of-the-art endoscopic procedures for pancreatic cancer. Future Oncol. 2016;12(17):2037–47. 10.2217/fon-2016-0123 27339021

[17] Leung D, Schwartz L. Imaging of neuroendocrine tumors. Semin Oncol. 2013;40(1):109–19. 10.1053/j.seminoncol.2012.11.008 23391118

[18] Gornals J, Varas M, Catalá I, et al. Definitive diagnosis of neuroendocrine tumors using fine needle aspiration-puncture guided by endoscopic ultrasonography. Rev Esp Enferm Dig. 2011;103:123–8. 10.4321/s1130-01082011000300003 21434714

[19] Bernacki KD, Betz BL, Weigelin HC, et al. Molecular diagnostics of melanoma fine-needle aspirates: a cytology-histology correlation study. Am J Clin Pathol. 2012;138(5):670–7. 10.1309/AJCPEQJW3PLOOZTC 23086767

